# The regulations of telomerase reverse transcriptase (TERT) in cancer

**DOI:** 10.1038/s41419-024-06454-7

**Published:** 2024-01-26

**Authors:** Mingdi Liu, Yuning Zhang, Yongping Jian, Liting Gu, Dan Zhang, Honglan Zhou, Yishu Wang, Zhi-Xiang Xu

**Affiliations:** 1 Key Laboratory of Pathobiology, Ministry of Education, Jilin University, Changchun, 130021 Jilin China; 2 Department of Urology, The First Hospital of Jilin University, Changchun, 130021 Jilin China

**Keywords:** Cancer genetics, Cancer

## Abstract

Abnormal activation of telomerase occurs in most cancer types, which facilitates escaping from cell senescence. As the key component of telomerase, telomerase reverse transcriptase (TERT) is regulated by various regulation pathways. *TERT* gene changing in its promoter and phosphorylation respectively leads to TERT ectopic expression at the transcription and protein levels. The co-interacting factors play an important role in the regulation of TERT in different cancer types. In this review, we focus on the regulators of TERT and these downstream functions in cancer regulation. Determining the specific regulatory mechanism will help to facilitate the development of a cancer treatment strategy that targets telomerase and cancer cell senescence.

**Figure:**
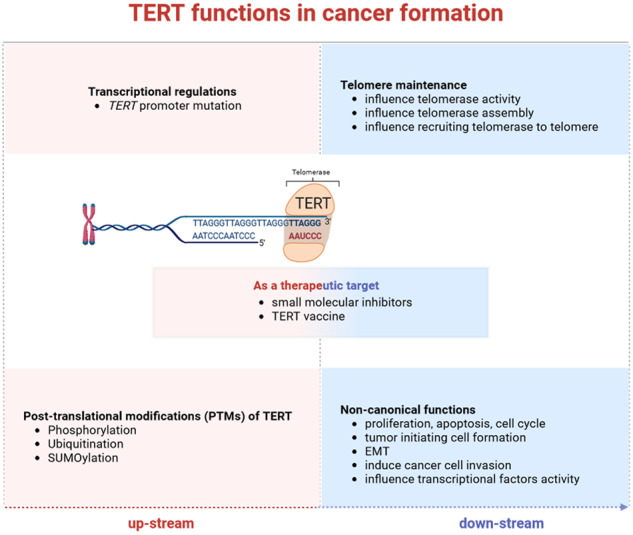
As the most important catalytic subunit component of telomerase, TERT is rapidly regulated by transcriptional factors and PTM-related activation. These changes directly influence TERT-related telomere maintenance by regulating telomerase activity in telomerase-positive cancer cells, telomerase assembly with telomere-binding proteins, and recruiting telomerase to the telomere. Besides, there are also non-canonical functions that are influenced by TERT, including the basic biological functions of cancer cells, such as proliferation, apoptosis, cell cycle regulation, initiating cell formation, EMT, and cell invasion. Other downstream effects are the results of the influence of transcriptional factors by TERT. Currently, some small molecular inhibitors of TERT and TERT vaccine are under research as a clinical therapeutic target. Purposeful work is in progress.

As the most important catalytic subunit component of telomerase, TERT is rapidly regulated by transcriptional factors and PTM-related activation. These changes directly influence TERT-related telomere maintenance by regulating telomerase activity in telomerase-positive cancer cells, telomerase assembly with telomere-binding proteins, and recruiting telomerase to the telomere. Besides, there are also non-canonical functions that are influenced by TERT, including the basic biological functions of cancer cells, such as proliferation, apoptosis, cell cycle regulation, initiating cell formation, EMT, and cell invasion. Other downstream effects are the results of the influence of transcriptional factors by TERT. Currently, some small molecular inhibitors of TERT and TERT vaccine are under research as a clinical therapeutic target. Purposeful work is in progress.

## Facts

- As the most important catalytic subunit component of telomerase, TERT is regulated by transcriptional factors and PTM-related activation.
- Telomere maintenance by regulating telomerase activity and assembly with telomere-binding proteins, as well as recruiting telomerase to telomere, are regulated by TERT in telomerase-positive cancer cells.
- The non-canonical biological function of TERT and its influence on different components of the tumor microenvironment in cancer cells, including angiogenesis, vascularization, EMT, proliferation, inflammatory factors, and immune response, are influenced by TERT.
- Small molecular inhibitors of TERT and TERT vaccine are under research as potential therapeutic targets.

## Introduction

Telomeres, the repetitive ends of chromosomes, comprise ca. 10–15 kb of tandem double-stranded 5′-TTAGGG-3′ repeats. Based on recent research by the ICGC/TCGA Pan-Cancer Analysis of Whole Genomes (PCAWG) Consortium, while telomere sequences comprise long stretches of 5′-TTAGGG-3′ (G-type) repeat arrays, there is increasing evidence that telomeric-associated sequences include variants—most commonly 5′-TGAGGG-3′ (G-type), 5′-TCAGGG-3′ (C-type), and 5′-TTGGGG-3′ (J-type) repeats, toward the proximal, subtelomeric regions [1]. These DNA sequences are coated with shelterin, a specialized protein complex that plays a fundamental role in regulating telomere protection [2–4]. Telomere structure and function serve to maintain chromosomal integrity and regulate cell division.

In most cancers, germline and stem cells, long-term proliferation requires telomerase activity to overcome the telomere shortening, which occurs with cell division. This shortening eventually arrests cellular growth, potentially providing an initial proliferative barrier to tumor formation in humans [5]. In adults, most stem cells exhibit telomerase activity, although at substantially lower levels. Although such reduced levels are sufficient for slowing down telomere shortening and increasing the replicative lifespan [6], they cannot prevent replicative senescence. Telomeres maintain DNA by acting on its 3′ overhang via the telomerase RNA component (TERC or TR) template; the telomerase adds single-stranded 5′-TTAGGG-3′ repeats to the lagging strand of the newly synthesized DNA. Finally, new primer RNA is added, and this is subsequently filled in with DNA polymerase alpha. In cancer, abnormal telomerase activation causes cancer cells to overcome cell growth arrest, resulting in cell immortalization [7].

Telomerase reverse transcriptase (TERT) is a key component of telomerase. Human TERT was first cloned in 1997 [5, 8–10], eight years after telomerase was first identified in HeLa cells [11]. The catalytic component of the telomerase holoenzyme complex comprises single molecules of TERT, WRAP53 (also known as TCAB1), two molecules of H/ACA ribonucleoprotein complex subunits DKC1, NOP10, NHP2, and GAR1, and a telomerase RNA template [12–14]. While TERT is rarely detected in normal human cells, except in embryonic cells, germ cells, and stem cells, TERC is universally expressed as a lncRNA in normal cells [5, 12, 15–17]. However, when acting as part of the telomerase holoenzyme [18], TERT can regulate telomere elongation, ultimately participating in tumor formation in a telomere-dependent or -independent manner.

Mutant TERT promoters, commonly detected in various types of cancer, induce TERT overexpression by recruiting transcription factors that do not normally regulate TERT gene expression and promoting abnormal telomerase activation [19–23]. Functionally, TERT affects cancer formation mostly by maintaining telomere length and enabling cells to avert cell senescence [24]. TERT has other non-canonical activity independently of its telomere-lengthening function, in particular, affecting the DNA repair response [25], gene transcription [24], and ubiquitin-proteasomal function [26, 27]. In this review, we focus on the regulators of TERT and the specific regulatory in cancer. These contents will help to facilitate the development of the cancer treatment strategy that targets telomerase and cancer cell senescence.

### Structural properties of TERT

TERT contains 1132 amino acids and comprises 4 telomerase-specific motifs, including the telomerase essential N-terminal (TEN) domain, the TERT RNA-binding domain (TRBD), the reverse transcriptase (RT) domain, and the C-terminal extension (CTE) [8, 28–30]. A new TERT domain, TRAP, was recently characterized via cryoelectron microscopy mapping of the *Tetrahymena* telomerase; TRAP is a large insertion of RT fingers [31, 32]. TERT and telomerase RNA (hTR) are composed of telomerase catalytic core of telomerase; TERT is associated with the pseudoknot/template (PK/t) domain and conserved regions 4 and 5 (CR4/5) of hTR. A H/ACA ribonucleoprotein (RNP) lobe is essential for telomerase biogenesis, which forms a telomerase holoenzyme with a TERT-related catalytic core [33].

Telomerase access to and activity at telomeres for telomere maintenance is tightly regulated. In normal conditions, a six-numbered protein complex, called shelterin, is composed of POT1, TPP1, TIN2, TRF1, TRF2, and RAP1 and protects the telomeric DNA ends. As a main component of shelterin, TPP1 has been implicated in telomerase recruitment to telomeres. Structurally, the TEN domain of TERT provides an anchor site for binding to telomeric DNA, while this binding initiates the primer-template interaction for telomere elongation. Furthermore, the TEN domain provides binding sites for TPP1 and contributes indirectly to telomerase activity by forming a complex with TRAP that stabilizes the active fold of TRAP, which participates directly in telomere repeat-addition processivity [30, 32]. TRBD interacts with the RT thumb subdomain, resulting in a closed ring-like tertiary structure with a large cavity at its center that binds to the primer-template duplex [34]. The fingers and palms of the TERT’s RT domain interact with the RNA backbone to place the template in the active site [35]. The RT motifs secure the hTR to the protein to maintain ribonucleoprotein stability and provide an active site for catalysis [36]. The CTE and TRBD domains of TERT interact simultaneously with the CR4/5 domain of hTR. Although the hTR binding sites are not active TERT sites, this interaction ensures the correct positioning of the TERT domain following TPP1 binding to the TEN domain, which allows the anchor site to engage the DNA substrate [37]. Moreover, TPP1, POT1, and TIN2 form a complex within Shelterin. POT1 binds to both the DNA substrate and TEN domain of TERT, forming a gate in front of the active telomerase site, which stabilizes DNA binding [38]. As a result, TPP1 and POT1 provide more space to accommodate the G-quadruplex structures formed by the telomeric-DNA product, reduce DNA dissociation during repeat-synthesis, and cooperatively increase telomerase repeat-addition processivity [5, 16, 37, 38].

## Effects on TERT in cancer formation

### Transcriptional regulation

It is suggested that the transcription of hTERT is the dominant step in the regulation of telomerase activity. Recurrent mutations and chromosomal rearrangements in TERT promoters are frequent in human cancers.

Alteration in the *TERT* gene includes genomic gains/amplifications, promoter rearrangements, and “hotspot” promoter mutations at a position relative to the transcription start site [39]. These alterations have been reported in multiple cancer types, especially *TERT* promoter mutations, which are associated with increased *TERT* expression [20, 21, 40–42]. In a pan-cancer study of somatic TERT promoter mutations and amplification, Sounak et al. found that among 30,733 specimens, almost 11.3% of cases with *TERT* promoter mutation were accompanied by 2.3% *TERT* amplification and 86.3% with no TERT amplification or promoter mutation [40]. It is indicated that TERT overexpression, caused by promoter mutation, is attributed to telomerase abnormal activation in cancer, although only a small portion of cancer patients are recognized as having TERT mutation.

hTERT promoter mutations are recognized in melanomas, while different types of cancer, including astrocytic glioma [43], sebaceous neoplasms [41], thyroid cancer [44], bladder cancers [45], and chondrosarcoma [46] have also been detected. The promoter mutation −124 C > T has been reported in almost half of patients with chondrosarcoma [46]; together, −124 C > T and −146 C > T are potentially important biomarkers in malignant melanoma linked to poor prognosis [42]. In 2020, members of PCAWG characterized the genomic footprint of the telomere mechanism and reported C228T or C250T promoter mutations in different cancer types [1], consistent with findings obtained in thyroid cancer and sebaceous neoplasms [41, 44]. However, TERT promoter mutations are not frequently detected in leukemias and colorectal cancers [47, 48]. Therefore, it has been hypothesized that in cancers originating in tissues with low self-renewal rates and limited ability to maintain tissue homeostasis, hTERT promoter mutations occur more often than those in cancer that already display high telomerase expression [47, 49]. Allelic DNA methylation within the *TERT* promoter further contributes to the regulation of TERT expression in cancer. Differential allelic expression occurs in almost 50% of cancer cell lines [50], which is more common than in human normal tissues.

Hypermethylation of the *TERT* hypermethylated oncological region enhances TERT expression when coupled with a hypomethylated proximal core promoter. However, this impact is abolished when the core *TERT* promoter is hypermethylated, which is often the case when *TERT* promoter mutations are present [51, 52].

Mechanistically, mutant *TERT* promoters enable “enhancer hijacking” near the *TERT* transcription start site [1, 53], which eventually leads to telomerase activation by abrogating transcription silencing of *TERT* [47]. In glioblastoma multiforme (GBM) cells, the NF-κB signal pathway selectively induces TERT expression with mutant TERT promoters, which is due to the p52-binding sites formation after specific mutation in TERT promoters [54]. In BRAF^V600E^-driven human cancer cells, including melanoma cell line A375, thyroid cancer cell lines BCPAP and 8305 C, and breast cancer cell line MDA-MB-231, MAPK/ERK signaling affects the binding capacity of GABP to mutant TERT promoter and eventually results in TERT activation. Specifically, the downstream factor Sp1 is phosphorylated, resulting in HDAC1 dissociation from the zinc finger DNA-binding domain of Sp1 and constantly promotes GABPA binding to the mutant *TERT* promoter [55]. Similarly, in BRAF^V600E^ papillary thyroid cancer (PTC) cells, MAPK pathway activation by the BRAF^V600E^ mutation upregulates E-twenty six (ETS) transcription factors, including *ETV1*, *ETV4*, *ETV5*, and *ELF3*, and increases TERT expression by binding to the ETS-binding site which generated by the TERT promoter mutations [56]. Phosphorylation of FOS, another downstream factor of *BRAF V600E*, promotes FOS binding to the GABPB 5′ UTR, constantly activating GABPB and promoting its binding to the mutant *TERT* promoter in PTC cells and melanoma cells [57, 58]. In summary, these studies demonstrate that *BRAF V600E* and *TERT* promoter mutations cooperatively upregulate *TERT* expression via the activation of the MAPK signal pathway dependent on FOS and GABP.

### Post-translational modifications (PTMs) of TERT

Following translation, PTM occurs via the addition of various chemical groups to one or more amino acid residues. PTM, which is especially common in cancer, can change the protein’s physicochemical properties by altering its spatial conformation and activity state, subcellular localization, and protein–protein interactions [59]. PTMs increase the biochemical diversity of TERT functions, thereby affecting telomerase regulation in cancer cells. TERT phosphorylation is the most important type, which plays an important role in regulating telomerase holoenzyme integrity. Thr249 [60], Ser824 [61], Ser227 [61], Ser457 [62], and Tyr707 [63] are known sites of phosphorylation by different kinases; the specific impacts are listed in Table 1.

**Table 1:** Functions and modulators of TERT post-translational modification.

Modification type	Mediator		TERT post-transcription sites			Downstream effects or observed functions	Models used in the study	References	
Phosphorylation		Akt		Serine 824,	enhances telomerase activity		SK-MEL 28		Kang, Kwon [61]
		Akt		Serine 227	promotes nuclear translocation of hTERT and		H1299		[64]
		Protein kinase C		Unknown	maintains telomerase holoenzyme integrity and regulates telomerase activity		OEC-M1, nasopharyngeal carcinoma-derived cell line, PMC42		[69]
		CDK1		Threonine 249	related to RdRP activity		HeLa		[60]
		Src kinase family (dephosphorylation via Shp-2)		Tyrosine 707	TERT export from the nucleus to mitochondria		293 T, human embryonic kidney cells		[63, 190]
		DYRK2		Serine 457	promotes ubiquitination of TERT for degradation		HeLa, 293 T		[62]
		MAPK		Unknown	Increases telomerase activity		A2780, HT-29		[82]
		c-Abl		Unknown	Inhibits telomerase activity and negatively regulates telomere length		293 T, MCF7		[85]
		BCR-ABL (BRC, breakpoint cluster region gene from chromosome 22, fuses with the ABL gene on chromosome)		Unknown	alters TERT location and reduces telomerase activity		K562, HL60		[87]
Ubiquitination		MKRN1		C-terminal domain of hTERT (residues 946–1132)	Induces TERT polyubiquitination		U2OS		[91, 92]
		HDM2		N-terminal lysine sites at residues 78, 94, 236, 339, and 348	Induces TERT polyubiquitination,		U2OS, HCT116, H1299, 293 T		[95]
		CHIP		Unknown	Promotes human telomerase reverse transcriptase degradation, promotes cytoplasmic accumulation, and inhibits nuclear localization without telomerase activity changing		H1299		[99]
		EDD–DDB1–VprBP E3 ligase complex		Unknown	inhibits telomerase via TERT degradation		MCF7, 293 T		[62]
SUMOylation		SUMO1, via CBX4 (SUMO E3 ligase)		Lysine 710 (predicted)	enhances migration and invasiveness		MCF7		[106]

In the human melanoma SK-MEL 28 cell line, Akt (also known as protein kinase B) phosphorylates TERT at serine 824 and 277, enhancing telomerase activity [61]. Akt inactivation via the phosphatidylinositol-Akt kinase pathway inhibitor wortmannin diminishes TERT phosphorylation and inhibits human telomerase activity [61]. In H1299 cells, phosphorylation at serine 227 by Akt is required for efficient nuclear translocation of hTERT and cellular immortalization [64]. Genistein, an isoflavone abundant in soy, has multiple molecular effects on cancer propagation [65]. In prostate cancer cells, genistein reduces telomerase activity by inhibiting Akt, thereby dephosphorylating TERT [66, 67]. The dietary component of genistein is a potential therapeutic molecule within a TERT-targeting cancer treatment strategy.

Protein kinase C (PKC), a serine/threonine kinase, participates directly or indirectly in various signal transduction pathways via target-protein phosphorylation [68]. PKC phosphorylation of TERT promotes the association between hsp90 and TERT, thus maintaining telomerase holoenzyme integrity [69]. The PKC activator (e.g., phorbol myristate acetate) enhances telomerase activity, whereas the PKC inhibitors (BIC, H-7, and bisindolylmaleimide I) inhibit it [69–71]. PKC isoenzymes *α*, β, δ, ε, and ζ are overexpressed in cancer patients and differentially correlated with high telomerase activity in different cancer types. In head and neck cancer cells, OEC-M1, PKC α, β, δ, ε, and ζ are involved in telomerase regulation through phosphorylation [69]. In nasopharyngeal carcinoma cells, among these isoenzymes, only PKC-ζ participates in activating telomerase activity [72]. In human breast cancer cell PMC42, PKC-α is implicated in TERT phosphorylation [73], while protein phosphatase 2 A (PP2A) directly dephosphorylates TERT, affecting telomerase assembly and disrupting its activity on the contrary [74]. These findings reveal the various functions of different PKC isoenzymes.

CDK1, a cell-cyclin-dependent serine/threonine kinase, plays an important role in telomere maintenance [75, 76]. During mitosis, CDK1 phosphorylates TERT at threonine 249, and this posttranslational modification is essential for tumor formation in pancreatic cancer and hepatocellular carcinoma [60]. hTERT phosphorylation at threonine 249 is positively associated with aggressive cancer phenotypes with high proliferative activity, high pathological grade, and severe nuclear atypia [77]. Phosphorylated hTERT expression is not associated with telomere length, suggesting that this phosphorylation might influence tumor formation dependent on RdRP activity rather than telomerase activity in the HeLa cell line.

Under oxidative stress, the Scr kinase family phosphorylates TERT at tyrosine 707, triggering TERT export from the nucleus to mitochondria in a GTPase Ran-dependent manner [63, 78]. In addition, H_2_O_2_ treatment downregulates mitochondrial TERT via phosphorylation by Src kinase in human embryonic kidney cells [79], which indicated that Scr kinase-related TERT phosphorylation could result in the translocation of TERT protein, which might influence telomerase activity both in cancer cells and human embryonic kidney cells.

Dual-specificity tyrosine-(Y)-phosphorylation-regulated kinase 2 (Dyrk2), also a cyclin-dependent kinase [80], binds directly to TERT, phosphorylating it at Ser457 [62], following which the Dyrk2–EDVP E3 ligase complex ubiquitinates TERT for degradation, downregulating it [62].

Hypoxia increases telomerase activity, possibly by activating the mitogen-activated protein kinase (MAPK) signal. In the A2780 and HT-29 cell lines, MAPK inhibitors diminish the upregulation of telomerase activity but do not influence basal telomerase activity [81]. In many cancers, inhibiting MAPK pathway effectors inhibits TERT mRNA expression [82]. However, evidence showing that MAPK directly phosphorylates TERT remains lacking.

c-Abl, a crucial prototypic non-receptor tyrosine kinase, is commonly activated by DNA damage and implicated in various cellular processes. c-Abl binds with and phosphorylates DNA-damage response proteins such as P73, and activates p53, often inducing cell death [83, 84]. Following exposure to ionizing radiation, c-Abl is activated by DNA damage and tyrosine phosphorylation of TERT increases; this is abolished by c-Abl deletion [85]. Following genotoxic stress, TERT phosphorylation inhibits telomerase activity and negatively regulates telomere length [85].

The oncogenic counterpart of c-Abl, the BCR-ABL fusion protein, causes certain human leukemias [86]. The translocation of chromosomes 22 and 9 results in the formation of the Philadelphia chromosome (Ph), the cytogenetic characteristic of chronic myeloid leukemia [87]. In this process, part of the breakpoint cluster region (BCR) gene on chromosome 22 fuses with the ABL gene on chromosome 9, forming BCR-ABL fusion protein with tyrosine kinase [88]. TERT expression and telomerase activity are higher in BCR-ABL positive cells (the K562 cell line) than BCR-ABL deficient cells (the HL60 cell line), as a result of TERT phosphorylation at tyrosine residues by BCR-ABL [87]. Gleevec inhibits TERT phosphorylation and downregulates TERT mRNA in BCR-ABL positive cells by inhibiting BCR-ABL kinase activity [88]. In BCR-ABL positive cells, Gleevec (imatinib), an inhibitor of receptor tyrosine kinase, induces telomerase translocation out of the nucleolus, indicating that TERT phosphorylation can influence telomerase activity by altering its location in cancer cells, consistent with other findings [64, 89, 90].

Ubiquitination of TERT, another form of PTM, also occurs in human cancers. Makorin Ring finger protein 1, encoded by *MKRN1*- a ubiquitin ligase, is a TERT-binding protein that regulates TERT degradation [91, 92]. The proteasome inhibitor MG132 reverses the loss of telomerase activity by promoting MKRN1 accumulation during cell cycle arrest [92]. Sphingosine kinase 2 generates lysophospholipid sphingosine 1-phosphate, a factor that binds to TERT and inhibits the interaction of TERT with MKRN1 [93]. Human double minute 2 (HDM2), an E3 ubiquitin-protein ligase, induces ubiquitination of target genes involved in tumorigenesis, including p53 [94]. As with MKRN1, HDM2 binds to TERT and induces TERT polyubiquitination [95]. In U2OS cells, HDM2 polyubiquitinates TERT principally at the N-terminus at five potential sites. Depletion of HDM2 strengthens cellular protective effects against apoptosis by stabilizing TERT [95]. HDM2 is also required in the p73-dependent relief of p53-mediated TERT suppression on a transcriptional level [96].

Polo-like kinase 1 (Plk1) is a Ser/Thr kinase that participates primarily in various aspects of mitotic events. Plk1 interacts directly with TERT, independently of its kinase activity, and enhances the stability of TERT by inhibiting its ubiquitin-mediated degradation by MKRN1 and HDM2, thereby increasing cancer cell telomerase activity [97, 98]. Hsc70-interacting protein (CHIP), which interacts with Hsc/Hsp70, is physically associated with TERT and downregulates telomerase activity via ubiquitin-mediated degradation, independent of Hsp90 binding. This degradation results in cytoplasmic accumulation of TERT, inhibiting its nuclear localization [99]. However, CHIP does not influence telomerase activity because the interaction between TERT and CHIP occurs in the cytoplasm, suggesting that CHIP may be associated only with intermediate or immature nonfunctional TERT [99].

Dyrk2 can directly phosphorylate TERT, which results in TERT degradation. This degradation is associated with the EDD-DDB1-VprBP E3 ligase complex, while Dyrk2 acts as a scaffolding protein for ubiquitination substrates [62].

Small ubiquitin-related modifier (SUMO) modification (SUMOylation) is a key regulatory modification of eukaryotic cells that may alter target protein activity, interactions, or longevity, ultimately influencing physiological processes, especially those in cancer cells [100–105]. SUMO1 directly SUMOylates TERT via CBX4, a SUMO E3 ligase. The SUMOylation of TERT enhances the migration and invasiveness of MCF7 cells [106].

## TERT-related regulations in telomere maintenance

**Fig. 1:**
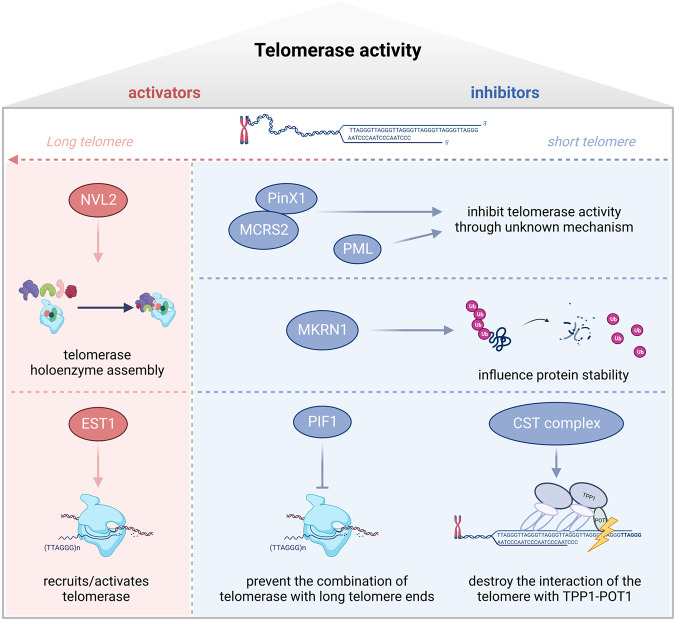
Activators and inhibitors targeting telomerase reverse transcriptase (TERT), involved in regulating telomerase activity. The activators include NVL2 and EST1 lengthen telomere by influencing telomerase holoenzyme assembly and activating telomerase, respectively. Some inhibitors such as PinX1, MCRS2, and PML inhibit telomerase activity through unknown mechanism. MKRN1 influences protein stability of TERT and decreases telomerase activity eventually. PIF1 could prevent the combination of telomerase with long telomere ends which leads to telomere shortening. CST complex which combined with CTC1, TEN1, and STN1 could destroy the interaction of the telomere with TPP1-POT1 and also leads to telomere shortening.

TERT is regulated by specific modulators, and these regulations influence the telomerase assembly, trafficking, blocking association, and mainly telomerase activity. Some positive regulators of telomerase promote telomere elongation by influencing telomerase holoenzyme assembly. In contrast, negative regulators inhibit telomerase activity, particularly by preventing telomerase from binding with the long telomere-end or with telomere-binding proteins (Table 2 and Fig. 1).

**Table 2:** Associating or binding partners of TERT and their effects.

Associating/binding partners of TERT	Effects on TERT/telomerase	Downstream effects or observed functions	Models used in the study	References
PinX1	Binds to TERT inhibitory domain	Inhibits telomerase activity and maintains PinX1-mediated TRF1 nucleolar localization	HT1080, GM847, PinX1^+/−^ C57mice and MEFs	[125–128]
MCRS2	Binds to TERT and forms a stable complex	Inhibits telomerase activity and shortens telomere	SMMC-7721	[129]
PML	Promotes TERT localized to PML nuclear bodies	Inhibits telomerase activity and shortens telomere	H1299, HepG2, U2OS, HFF	[130]
NVL2	Promote telomerase holoenzyme assembly	Upregulates telomerase activity	HeLa	[110]
hEST1A and hEST1B	Activating or recruiting telomerase to the telomere	Lengthens telomere	HeLa,293	[111, 112]
TPP1	Interacts with the TEN domain of TERT	Telomerase then extends the G-rich strand of the telomere via TPP1–POT1 complex-promoted translocation of telomerase		[113–119]
PIF1	Prevent telomerase from combining with the long telomere ends	Inhibit telomerase activity	yeast cells	[120, 121]
PIF1		Influences proliferation, apoptosis, and cell cycle independent of telomerase activity	Hela, SiHa, Ca-Ski, C-33A	[122, 123]
ATF7	Binding to TERT via Ku70	Mediates telomere shortening independent of telomerase activity	*Atf7*^*−/−*^ mice model MEFs, HeLa	[191]
BRG1		Forms TERT-BRG1-nucleostemin complex drives tumor-initiating cell formation	HeLa, MCF7, BJ, 293 T, HA1ER	[131, 132]
c-MET		Promotes the expression of mesenchymal markers, including vimentin and N-cadherin	A549, H1299	[135]
NF-κB p65		Influence NF-κB-dependent gene expression such as *IL-6*, *TNF-α*, and *MMPs*, which are critical for cancer progression	HeLa	[136, 137]
MYC		Stabilize MYC protein and influence MYC-related promoters such as *EIF2A* and *NCL*	P493, EμMYC B cell, lymphomas, 293 T	[138]
Sp1		Activate Sp1-related gene transcription, including *VEGF* and *DNMT3B*	293 T, Human umbilical vein endothelial cells, HCC-derived cell lines PLC/PRF/5 and HUH-7	[139, 141]

### Factors that influence telomerase holoenzyme assembly

**Fig. 2:**
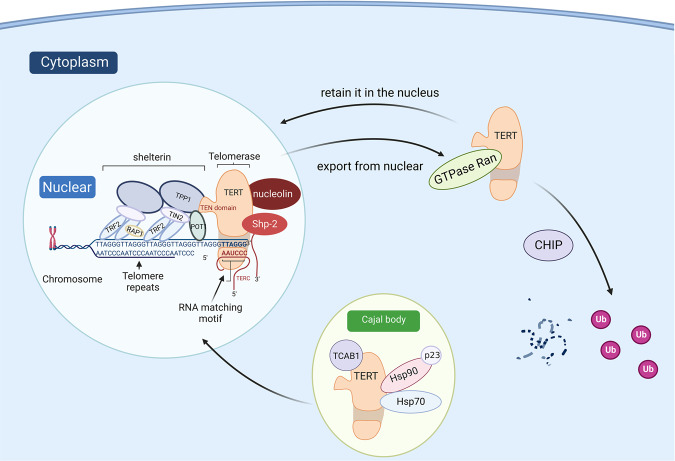
The transportation of TERT. Transportation of TERT from the Cajal body to nuclear following telomerase construction due to Hsp90 and p23 through the activation of TCAB. Other factors participating in TERT translocations are described, and the interaction between TERT and shelterin complex is shown.

Different domains and unique spatial structures provide a structural basis for TERT to assemble telomerase holoenzymes. Therefore, modulators that contribute to the intracellular distribution of TERT also influence this tightly regulated process. The 70-kDa heat shock protein (HSP70), a ubiquitous molecular chaperone that controls various cellular protein-folding and remodeling processes [107], transiently binds to TERT in the absence of TR. The 90-kDa heat shock protein (HSP90), accompanied by chaperone p23, binds specifically with TERT and ensures its correct assembly with the template RNA [108]. HSP90 and p23 load TERT into the Cajal body, generating an enzymatically active telomerase complex. Once an active assembled complex is formed, the combination of HSP70 and TERT is abolished [108] and Telomerase Cajal body protein 1 (TCAB1) then binds to TERT and facilitates telomerase translocation from the Cajal bodies into the nucleoplasm, where TPP1 facilitates the recruitment of the complex to the telomere (Fig. 2) [12, 109].

In HeLa cells, Nuclear valosin-containing protein-like (NVL), which was first recognized as an interactor with TERT in yeast, could interact with TERT and is positively associated with telomerase holoenzyme assembly. Knockdown of endogenous NVL2 downregulates TERT, reducing telomerase activity [110]. This suggests that NVL2 is essential for telomerase enzymatic activity.

### Factors that influence the recruitment of telomerase to the telomere

After telomerase holoenzyme assembly, the recruitment of telomerase to the telomere is a limiting step to promoting telomere addition. The modulators of TERT also participate in this critical process. hEST1A and hEST1B are human homologs of the yeast telomerase subunit Est1p, which is a functional factor in telomere maintenance. hEST1A and hEST1B bind to TERT independently of the RNA subunit, participating in telomere maintenance by activating or recruiting telomerase to the telomere [111, 112].

During the S phase of the cell cycle, telomerase is recruited to the telomere via the interaction of TPP1 with the TEN domain of TERT; telomerase then extends the G-rich strand of the telomere via TPP1–POT1 complex-promoted translocation of telomerase. In the late S/G2 phase, the CST complex (comprising CTC1, TEN1, and STN1) restricts the interaction between telomerase and TPP1–POT1, preventing telomerase from accessing the newly synthesized G-rich telomere strand, thus enabling it to bind to telomeric single-stranded DNA [113–119].

The PIF1 family helicases, which have multiple roles in eukaryotes, negatively regulate telomerase activity by preventing it from combining with the long telomere ends [120, 121]. In addition, PIF1 binds directly to TERT and influences proliferation and apoptosis independent of telomerase activity, although the mechanisms remain unclear in cervical cancer cells [122, 123].

Activating transcription factor 7 (ATF7), the primary factor in stress-induced telomere shortening, binds to TERT and leads to the release of ATF7 and telomerase from telomeres, eventually resulting in telomere shortening [124]. However, in HeLa cells, shRNA-induced alteration of ATF7 expression did not affect telomerase activity, which indicated a telomerase-independent telomere length regulation [124].

### Factors that influence telomerase activity

As the main component subunit of telomerase, the regulations of TERT result in changes in telomerase activity. The oncogene PinX1, first identified in a yeast two-hybrid screen as a TRF1-binding protein, is a major haploinsufficient tumor suppressor essential for maintaining telomerase activity and chromosome stability [125]. PinX1 binds to the 74 aa C-terminal fragment of TERT in its telomerase inhibitory domain, inhibiting telomerase activity and thereby inducing telomere shortening [126]. PinX1–TERT binding maintains PinX1-mediated TRF1 stability and influences the TRF1 nucleolar localization, which is important in preserving telomere integrity [127, 128].

MCRS2, a novel isoform of human micro-spherule protein 1 (MCRS1), interacts with PinX1. MCRS2 binds to TERT, forming a stable complex that inhibits telomerase activity, thereby shortening the telomere [129].

The gene encoding promyelocytic leukemia (PML), a tumor suppressor primarily expressed in the nucleoplasm, interacts directly or indirectly with TERT in HepG2, U2OS, and HFF cell lines, negatively regulating telomerase activity and eventually resulting in telomere shortening [130].

## TERT-related regulations facilitate oncogenesis independent of telomerase

**Fig. 3:**
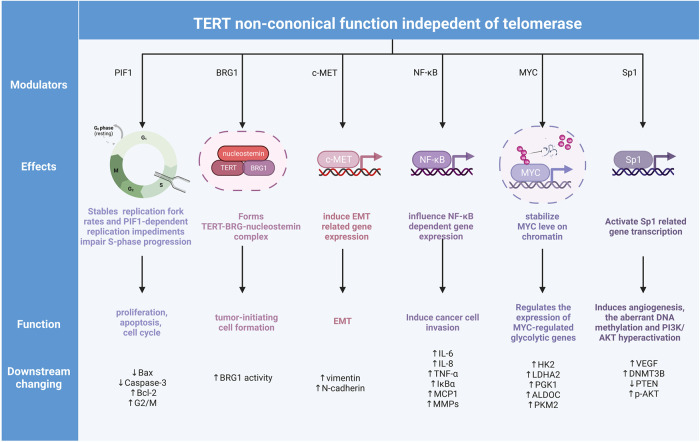
Non-canonical TERT function in cancer regulation. Instead of telomerase regulation, TERT also participate in proliferation, apoptosis, cell cycle controlling, tumor-initiating cell formation, EMT, cell invasion, and the regulation of transcriptional factor-related gene expression changing processes. These changings in cancer also influence the components in tumor microenvironment.

TERT exhibits non-canonical functions involved in tumor oncogenesis independent of telomerase activity, which is detailed in the following sections (Table 2 and Fig. 3).

### Factors that influence telomere maintenance independent of telomerase

In human cancer cell lines, TERT physically forms a complex with BRG1, a SWI/SNF-related chromatin remodeling protein, and nucleostemin, a nucleolar GTP-binding protein. The TERT–BRG1–nucleostemin complex drives tumor-initiating cell formation by regulating BRG1 activity rather than contributing directly to telomere maintenance [131, 132].

### Factors that influence different components of the tumor microenvironment

According to the heterogeneity of different types of cancers, the tumor microenvironment (TME) is composed of neoplastic, stromal, endothelial, and infiltrating immune cells, and the interactions of these elements determine the pathological process in cancer progression [133, 134]. Previous studies have shown that the interactions between TERT and other factors, including c-MET [135], nuclear factor κB (NF-κB) p65 [136, 137], Myc [138], and *Sp1* [139], influence downstream gene expression and signal transduction. These telomere-independent activities contribute to the regulations of other cell processes in TME, including angiogenesis, vascularization, epithelial-to-mesenchymal transition (EMT) [135, 140], and inflammation [136, 137], eventually promoting cancer cell proliferation [122].

#### Effects on blood vessels: angiogenesis and vascularization

In human gastric tumor samples, TERT expressions are positively associated with VEGF expressions. Mechanistically, *Sp1*, the transcription factor of VEGF, interacts with TERT and facilitates angiogenesis in cancer via VEGF activation [139]. In hepatocellular carcinoma cells, TERT binding to *Sp1* stimulates DNMT3B transcription and induces aberrant cancer-specific global DNA methylation and AKT hyperactivation [141]. The activation of AKT modulates the increased expression of VEGF and activates angiogenesis both in normal tissues and in cancers [142]. The binding of TERT to MYC in cells with high MYC expression can stabilize MYC protein by inhibiting MYC ubiquitination, thereby activating the downstream targets of MYC, such as *EIF2A* and *NCL* [138]. In breast cancer cells, VEGF expression is increased by c-MYC and eventually induces tumor angiogenesis [143]. TERT also regulates MMP expression, especially MMP9, in an NF-κB-dependent manner [137]. MMP9 knockdown inhibits angiogenesis in prostate cancer cells via the release of angiostatin, which serves as a key regulator of angiogenesis [144]. Besides, in an NSCLC mouse model, TERT deficiency decreased the positive signal of PanCK/CD31 double staining and CD34 staining, suggesting the involvement of TERT in vascularization [145].

#### Effects on mesenchymal tissue

In A549 and H1299 lung cancer cells, hTERT overexpression upregulates c-MET and promotes the expression of mesenchymal markers, including vimentin and N-cadherin [135]. In the NSCLC mouse model, TERT deficiency inhibits the expression of c-MET and reduces EMT by influencing the expression of mesenchymal markers, including vimentin and fibronectin [145]. Similarly, a *TERT* promoter variant enhances EMT in prostate cancer and promotes the development of castration-resistant prostate cancer [140]. In HPV-immortalized cervical cancer cells, overexpression of TERT alters EMT characteristics [146]. The authors showed that TERT is a critical factor in the EMT process in cancer progression.

#### Effects on inflammatory factors and immune response

The NF-κB family comprises inducible transcription factors with important roles in cancer progression [147] and acts as a pro-inflammatory transcription factor involved in the regulation of inflammation [147, 148]. TERT binding to NF-κB (also known as p65) is required for the promotion of NF-κB-dependent gene expression such as IL-6 and TNF-α [136]; these factors are critical for inflammation in cancer progression. In an NSCLC mouse model, TERT promotes lung inflammation by influencing the expression of *Ifng*, *Tnf*, *Il10*, and *PD-1*, markers of inflammation, and tumor immunosuppression [145].

TERT also affects the immune system and is considered a self-antigen constitutively expressed in many tumors, with potential therapeutic value for anticancer immunotherapy [149]. TERT is recognized by the adaptive immune system; in particular, CD4^+^ and CD8^+^ T cells recognize TERT as short peptides processed inside the cell before being exported to and presented at the cell surface by major histocompatibility complex I/II molecules, which induces an adaptive immune response associated with the inhibition of tumor growth [150–154]. These findings provide the theoretical basis for the discovery and the clinical application of the TERT vaccine in cancer treatment.

## The current and future pharmacological strategies targeting TERT or telomerase

Owing to their good anti-cancer properties, TERT and telomerase are used as therapeutic targets for telomerase-positive cancer. Small molecule inhibitors and telomerase-targeted vaccines are the most frequent types in drug development.

TMPyP4, RHPS4, BRACO-19, and telomestatin are typical ligands that fold the single-stranded telomeric DNA (the telomeric substrate) into a four-stranded quadruplex structure, inhibiting telomerase catalytic activity [155]. In humans, TMPyP4 downregulates TERT transcription [156], inhibiting telomerase activity and TERT expression [157, 158]. However, the suppression proliferative of RHPS4 in glioblastoma stem-like cells is independent of telomeric dysfunction [159], which indicates a differential influence in cancer treatment. Significantly, a high level of G-rich DNA exists throughout the genome, especially in the promoter regions of oncogenes. Therefore, G4 ligands have several risks when used in telomerase inhibition and may have a significant off-target effect [160–162].

6-thio-2’-deoxyguanosine (6-thio-dG, also named THIO) is a nucleoside analog that could induce telomere dysfunction by incorporating into telomeric DNA in telomerase-positive cells [163]. In NSCLC xenografts, 6-thio-dG treatment reduces tumor growth by increasing telomere damage [145]. The therapeutic effect has also been validated in Gliomas [164] and Melanoma [165, 166] both in vivo and in vitro. Ilgen Mender et al. found that 6-thio-dG mitigates chemotherapy resistance to ECFG inhibitors and is commonly used in chemotherapy combinations (e.g., gemcitabine and cisplatin) in NSCLC cell lines [167]. In xenografts from neuroblastoma cell lines, pediatric high-risk group-3 medulloblastoma xenografts, and an orthotopic patient-derived xenograft model of diffuse intrinsic pontine glioma, 6-thio-dG inhibits tumor growth by inducing telomere dysfunction, which showed a promising approach to mitigate therapy-resistant telomerase-positive pediatric brain tumors [168, 169]. Similarly, 6-thio-dG significantly improves the efficacy of the combination of anti-PD-L1 and anti-VEGF based on CD8^+^ T cells in Hepatocellular Carcinoma [170], consistent with earlier research [171].

Directly targeting active TERT sites is a more popular therapeutic strategy than using compounds that inhibit TERT DNA-binding [172, 173]. 2-[[(E)-3-naphthalen-2-ylbut-2-enoyl] amino] benzoic acid (BIBR1532) non-competitively binds to the active sites of TERT, inhibiting its telomerase activity [24, 30]. Amin et al. found that short-term BIBR1532 impairs cellular proliferation in a zebrafish model, which is a potential anticancer treatment strategy [174]. Recent research on new BIBR1532-based analogs, designed to overcome ineligible pharmacokinetics, showed greater antitumor activity in the Ehrlich carcinoma model [175].

Imetelstat (GRN163L), a specific telomerase inhibitor that directly binds to the TR component in the catalytic site of the telomerase enzyme, has been used in a clinical study [176]. Imetalstat can induce cell death and improve the treatment efficacy of chemotherapy in patient-derived xenografts of Acute myeloid leukemia (AML) [177, 178]. A phase II study of lower-risk myelodysplastic patients reported meaningful effects with Imetelstat [179], and a phase III study of lower-risk myelodysplastic syndromes who have relapsed or are refractory to erythropoiesis-stimulating agents reported that approximately 40% patients achieved transfusion independency at least 8 weeks [180]. Therefore, almost 91% of patients had grade 3–4 treatment-emergent adverse events [180]. These results indicate that telomerase-targeting therapy has considerable prospects but warrants further investigations to reduce adverse drug reactions.

Telomerases are HLA class-I antigens, hence TERT-derived vaccines have also been considered as an approach to stimulate immune response in targeting cancers [181]. To date, GV1001 single-use [182] or combined with HR2822 [183], UV1 [184] single-use or combined with Ipilimumab (checkpoint inhibitor) [185], Vx-001 [186], and Gx-301 [187] have undergone clinical trials in patients with different types of cancer. Based on CD4^+^/CD8^+^ T cell recognition, the TERT vaccine presents a promising therapeutic efficacy in a proportion of cancer, although its safety and universal applicability in different types of cancer requires further research and verification [153, 188, 189].

## Conclusions and perspectives

Telomerase reactivation is essential for cancer progression, but it does not occur during normal cell senescence. Telomerase-mediated stress responses in cancer result in telomere escape from shortening with every division. As the most important catalytic subunit component of telomerase, TERT is regulated by various regulation pathways, including transcriptional factors, PTM-related activation, and inhibition by co-interacting proteins.

The most common *TERT* gene changing is promoter mutant, which results in the binding of the ETS family transcription factor on “enhance hijacking” near the TERT transcription start sites. As for PTMs of TERT protein, phosphorylation has been extensively studied, and ubiquitination and SUMOylation have been reported in various cancer cells. The phosphorylation of TERT mainly results in telomerase activity changing by influencing the stability of TERT with the regulation of ubiquitination, maintaining telomerase holoenzyme integrity, and changing the translocation of TERT in cancer cells between nuclear and cytoplasmic. Besides, a small portion of the phosphorylation of TERT does not influence telomerase activity more than RdRP activity, which indicates that the functions of TERT in cancers should be considered.

The modulators of TERT, which regulate telomerase assembly, trafficking, blocking association, especially telomerase activity have been well-researched in recent years. Some positive regulators of telomerase promote telomere elongation by influencing telomerase holoenzyme assembly, recruiting telomerase to telomere, and stabilizing TERT protein. Negative regulators inhibit telomerase activity, particularly by preventing telomerase from binding with the long telomere-end or with telomere-binding proteins. Besides, the modulators that induce the non-canonical function of TERT that regulates gene expression by some transcription factors, such as c-MET, NF-κB p65, Myc, and Sp1, also participate in cancer progression.

To date, various small molecular drugs, including G4 ligands representative of TMPyP4 and telomerstatin, 6-thio-dG, BIBR1532, Imetelastat, and TERT vaccine, have been investigated. However, due to its limited effect and undetermined drug safety, further research remains warranted before clinical use.

To summarize, the TERT modulators discussed in this review provide potential targets for cancer treatment. Interventions targeting atypical telomere-related molecules influence cancer-associated cellular processes, exerting synergistic anticancer effects. However, further research remains warranted to identify novel treatment strategies.
